# Elevated vitamin D levels in diurnally-active female fruit bats

**DOI:** 10.1016/j.heliyon.2024.e38973

**Published:** 2024-10-04

**Authors:** Ofri Eitan, Maya Weinberg, Nirit Lavie Alon, Sahar Hiram-Bab, Yuval Barkai, Reut Assa, Adi Rachum, Omer Yinon, Yossi Yovel

**Affiliations:** a School of Zoology, Faculty of Life Sciences, Tel-Aviv University, Tel Aviv, 6997801, Israel; b Society for the Protection of Nature in Israel, Tel Aviv, 6618602, Israel; c The Steinhardt Museum of Natural History, National Research Center for Biodiversity Studies, Tel-Aviv University, Tel Aviv, 6997801, Israel; d Faculty of Medicine, Tel-Aviv University, Tel-Aviv, 6997801, Israel; e Sagol School of Neuroscience, Tel-Aviv University, Tel Aviv, 6997801, Israel; f School of Mechanical Engineering, Faculty of Engineering, Tel-Aviv University, Tel Aviv, 6997801, Israel

## Abstract

Animal species have evolved to enhance their survival by focusing their temporal activity on specific parts of the diurnal-nocturnal cycle. Various factors, including inter-specific competition and anti-predator behavior, as well as anthropogenic effects like light pollution, have prompted some species to expand or shift their temporal niches. Our study focuses on the temporal niche shift of the Egyptian fruit bat (*Rousettus aegyptiacus*) to diurnal activity in Israel.

Through an extensive citizen-science study, we assessed the distribution of these bats’ diurnal activity across Israel. We also documented the sex and age of bats from a colony known for its diurnal activity and collected blood samples from them for metabolic analysis.

Our findings indicate that the shift toward daytime activity predominantly takes place in urban settings and is mostly exhibited by females. We found a significant physiological effect of this temporal shift, namely: diurnal bats’ vitamin D levels were significantly higher, and their parathyroid hormone (PTH) levels were significantly lower than those of nocturnal bats.

We suggest that the reproductive metabolic demands of female bats might be a key factor driving this shift to diurnal activity. We hypothesize that the increase in vitamin D, derived from sunlight hours, might play a crucial role in regulating calcium homeostasis, thus contributing to the bats’ physiological needs during the reproduction season.

## 1 Introduction

Many animals have adapted to optimize their fitness by exhibiting activity during specific parts of the day-night cycle, including being diurnal, nocturnal, or crepuscular. These adaptations include a range of behavioral, physiological, and sensory changes [[1], [2], [3], [4]]. Various factors, such as food availability, temperature, predation risks, and competition [5,6], can affect the choice of the diel segment during which animals are active.

While most mammals are nocturnal, which offers advantages such as reduced risk of predation, this lifestyle also presents several challenges. Nocturnal animals face limited food resources, and increased competition for these resources [7,8]. Nevertheless, various factors have prompted certain species to expand their ecological niches or even undergo shifts in their temporal niches. Some of these factors include changes in environmental conditions, inter-specific competition, and anti-predator behavior, as well as anthropogenic effects like light pollution [1,2,[9], [10], [11], [12], [13]].

While diel plasticity is well-documented at the population level, shifts within specific segments of the population, such as age- [14] or sex [15]-dependent changes, are relatively rare. For example, it has been observed that juvenile Atlantic salmon (*Salmo salar*), when preparing to migrate, expand their activity into diurnal periods in order to access more food and thereby grow faster [14]. Another example of a sex-dependent temporal niche shift was demonstrated in a lab experiment: female common voles (*Microtus arvalis*) exhibited a switch to diurnal activity during lactation in response to elevated ambient temperatures [16]. In bats, it has been shown that male *Hipposideros ruber* bats on São Tomé Island display diurnal activity [15], while female sac-winged bats (*Saccopteryx leptura*) on Gorgona Island have been reported (though not quantified) to exhibit diurnal activity during the peak reproduction season in July [17].

A major environmental constraint for nocturnal mammals is that of lack of exposure to the sun and, consequently, to UV-B radiation, which may result in vitamin D deficiency [18]. Vitamin D is an evolutionarily conserved hormone that has many functions, including the regulation of calcium and phosphorus homeostasis [19], and bone metabolism homeostasis [19], but also brain development [20,21] and immune functioning [[22], [23], [24], [25]]. It can be synthesized by most mammals when exposed to sunlight, particularly to UV-B radiation [[26], [27], [28]]. The process involves the conversion of 7-dehydrocholesterol (7-DHC) to vitamin D3 upon penetration of solar UV-B radiation through the skin [29]. The D3 is later metabolized in the liver to 25-hydroxyvitamin D [25(OH)D]. The kidneys then hydroxylate 25(OH)D to its active form, known as 1,25-dihydroxyvitamin D [1,25(OH)2D] or calcitriol [[26], [27], [28]]. The production of the active form of vitamin D is regulated by the levels of the parathyroid hormone (PTH), calcium, and phosphorus in the body [29].

While vitamin D can be obtained from some food sources such as fish and mushrooms, most foods do not contain adequate levels of this vitamin [28,29]. Insufficient dietary intake of vitamin D or limited exposure to sunlight can lead to vitamin D deficiency, which in turn affects the body's ability to absorb dietary calcium [28,29]. When vitamin D levels are optimal, the body can absorb about 30–40 % of dietary calcium. However, in cases of vitamin D deficiency, this calcium absorption rate drops to approximately 10–15 %, which can lead to a decline in bone mineral density (BMD) [29].

When vitamin D or calcium concentrations are low, there is an increase in the serum levels of PTH [24,[30], [31], [32]]. PTH is synthesized by the parathyroid gland, and its main function is to maintain calcium homeostasis in the body [24,31]. The parathyroid gland produces more PTH, which in turn increases the number of mature osteoclasts (the bone cells that are responsible for degrading bone tissue) leading to bone resorption, which can result in weakened bones and an increased risk of fractures in bats and other mammals, including humans [24,29,[33], [34], [35]]. Studies have shown that people with vitamin D deficiency often have high PTH levels and suffer from low bone mineral density [[29], [30], [31], [32]].

Pregnancy and lactation are highly energetically demanding [36]. To meet these increased energy demands, females may cope by increasing their food intake [[36], [37], [38]]. There is a notable reproduction cost linked to pregnancy and lactation—a heightened need for calcium. Calcium plays a crucial role during these periods, as pregnant and lactating females transfer calcium, which is vital for their own bone metabolism, to their fetuses and newborns [33,38,39].

Bats, which comprise the second-largest mammalian order, containing ∼1500 species worldwide, are predominantly nocturnal, although some species are also diurnal [15,17,[40], [41], [42], [43], [44], [45], [46]], particularly on islands where it has been hypothesized that the absence of natural predators has allowed them to expand their temporal niche [17,47,48]. Studies have shown that several species of fruit-eating bats (family Phyllostomidae) have low levels of serum vitamin D, whereas fish-eating bats have high vitamin D levels due to their diet [28,49]. Exposure to sunlight has been shown to increase vitamin D levels in captive colonies of two fruit bat species (family Pteropodidae), such as the Egyptian fruit bat (*Rousettus aegyptiacus*) [26,50]. Based on these findings, it has been suggested that, in captivity, supplementary vitamin D could be important to enable lactating female Egyptian fruit bats to meet their calcium demands [26].

Female Egyptian fruit bats in temperate areas (including Israel) exhibit two reproduction seasons, giving birth in March and August, in contrast to females in the tropical zone, which reproduce once a year [[51], [52]]. The duration of gestation is approximately 4 months, resulting in one offspring, and the lactation period lasts around 80 days [[51], [52]]. For those that reproduce twice a year, this places significant metabolic and energetic demands on them. During gestation, pregnant females’ energy intake is increased by 35 %, while during lactation the metabolic energy intake is substantially higher, with an 80 % increase compared to non-pregnant females [53]. One mechanism by which to cope with this energetic demand is that of decreasing the resting metabolic rate. Females in their late pregnancy state exhibited a decrease of 19 % in metabolic rate compared to non-reproductive females [53]. The main strategy, however, is that of an increase in food consumption [53]. It has been shown that lactating females of several bat species exhibit increased foraging duration, emerge earlier, and fly longer distances than males, pregnant females, and non-reproductive females [54,55].

It has recently been shown that, despite being nocturnal, some Egyptian fruit bats are active during the day in Israel, foraging and drinking from artificial pools in Tel Aviv [42]. In our study, we investigated the temporal niche shift of Egyptian fruit bats by studying the activity of a wild colony known for its daytime activity [42]. The colony roosts in the Dizengoff Shopping Center's parking lot (hereafter DC) in central Tel Aviv. We measured serum levels of 25(OH)D and PTH in both diurnal and nocturnal bats. We hypothesized that vitamin D levels would be higher in diurnal Egyptian fruit bats compared to nocturnal ones, resulting in lower PTH levels. This might explain why reproductive female Egyptian fruit bats, having expanded their activity to daylight hours, potentially enhance vitamin D synthesis, improve calcium metabolism, and employ an alternative coping mechanism to meet the high metabolic and calcium demands associated with reproduction.

## 2 Results

### 2.1 Diurnal population assessment

#### 2.1.1 Surveying diurnal bat distribution in Israel

**Fig. 1:**
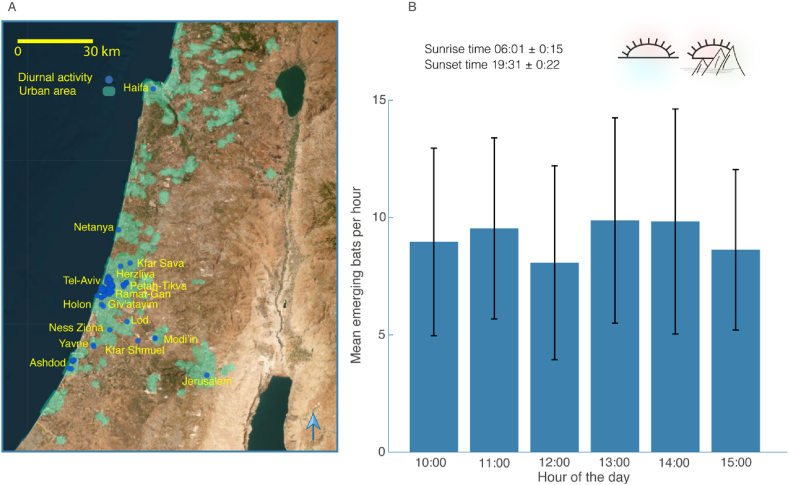
– Diurnal bats' distribution and activity patterns. (A) The diurnal activity of Egyptian fruit bats extends to urban environments throughout Israel. Observations provided by citizen-science reports have revealed that the diurnal activity of these bats is not confined solely to Tel-Aviv; rather, it is widely distributed across various urban areas. Confirmed observations are in blue, and the green overlay shows urban areas (Hazav project, Ministry of Transportation). (B) Bats do not show a specific preferred time for their diurnal activities. The bars represent the average number of bats emerging per hour ± SD, over a period of 21 days, with a total of 604 emergence events. The sunrise and sunset times are presented as the mean ± SD for these 21 days. (For interpretation of the references to color in this figure legend, the reader is referred to the Web version of this article.)

The daytime activity of Egyptian fruit bats has been reported in urban environments across Israel (Fig. 1A and Suppl. Table 1). Based on a citizen-science survey, we verified a total of 322 observations (and a total of 432 individuals) of diurnal bats from five districts incorporating 16 cities. Tel-Aviv is the city where this phenomenon has been best described. In total, there were 61 validated observations outside of Tel Aviv, suggesting that the phenomenon of daytime activity also occurs in additional areas.

#### 2.1.2 Diurnal bats' emergence time from the colony

We conducted observations at the entrance to the shopping center suppliers’ parking lot, where the DC colony is located, and documented the emergence times of bats throughout the day (Fig. 1B). Observations were carried out from 10:00 to 15:00, for a total of 21 days, spread over 14 weeks, including both weekdays and weekends when traffic was minimal to none (see Methods). We observed a total of 604 bats emerging, with no evident preference for a specific hour of emergence, either on weekdays or weekends. The time of day and type of day (weekday vs. weekend) did not significantly correlate with the frequency of exits (mixed-effect GLM - GLMM - with the number of observations per hour set as the explanatory parameter, the hour of activity and day type as a fixed-effects, and the observation date as a random effect, p = 0.34 for the activity hour and p = 0.75 for the type of day, df = 72).

#### 2.1.3 Sex and age ratio activity evaluation

**Fig. 2:**
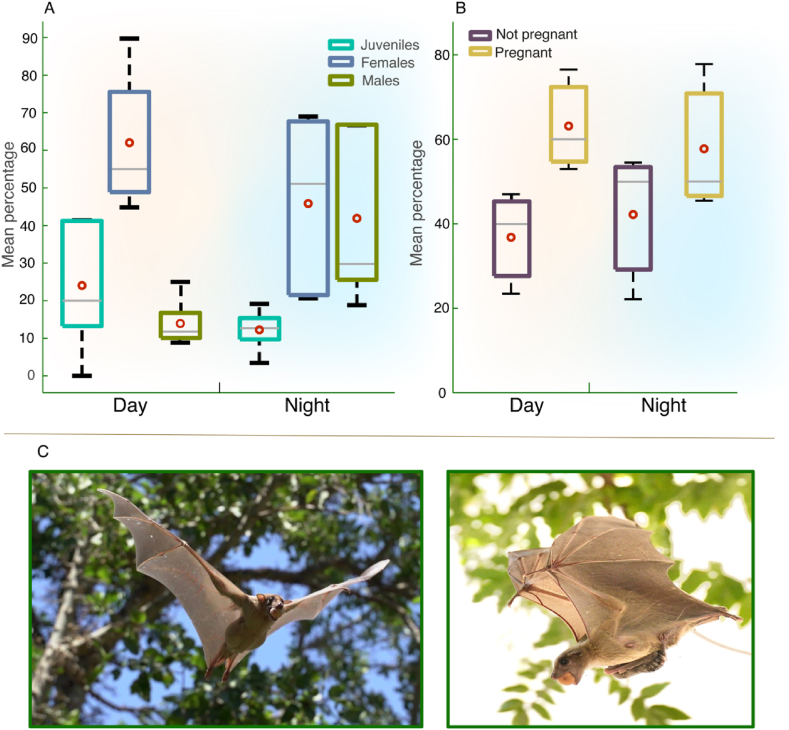
Sex and age-dependent diel activity – (A) Diurnal bats are mostly female. Box plots show the median and quartiles; The mean percentage represents the average percent per day in each category, based on a sample of 431 bats observed throughout five non-sequential days and nights (each one a 24-hr period). Orange shading depicts diurnal activity, and turquoise shading depicts nocturnal activity. Red circles depict the mean values. (B) There was no significant difference in the proportion of diurnal and nocturnal pregnant females. The mean percentage represents the average percent per day in each category, based on a sample of 81 bats observed throughout three non-sequential days and nights (each one a 24-hr period). Orange shading depicts diurnal activity and turquoise shading depicts nocturnal activity. Red circles depict the mean values. (C) Female Egyptian fruit bats engaging in daytime foraging, including during pregnancy (left) and while carrying a pup (right). (For interpretation of the references to color in this figure legend, the reader is referred to the Web version of this article.)

We captured bats emerging from and returning to the DC colony during five pairs of consecutive days and nights over a period of six months (see Methods). A total of 275 bats were caught at night (after sunset) and 156 during the day (2.5–5.5 h after sunrise). We documented the sex and age of these bats (Fig. 2A and Suppl. Table 2). Most of the daytime-active bats were adult females, accounting for 62.0 ± 18.2 % (mean ± SD, hereafter) vs. only 13.9 ± 6.0 % adult males and 24.0 ± 17.5 % juveniles. In contrast, during the night activity was more evenly distributed, with females accounting for 45.8 ± 23. 6 % of the captures, males for 41.9 ± 22.7 %, and juveniles for 12.2 ± 5.6 %. Diurnal activity was thus much more prevalent in females. (GLMM with sex as the explanatory parameter, activity time (day or night), as a fixed effect, and the capture date as a random effect, p < 0.02 for activity, df = 357). There was no significant difference in diurnal and nocturnal juvenile activity (GLMM with age as the explanatory parameter, diel activity as fixed effect, and the capture date as random effect p = 0.2, df = 429).

#### 2.1.4 Reproduction state assessment

We captured bats emerging from and returning to the DC colony during three pairs of consecutive days and nights (see Methods). A total of 32 females were caught at night (after sunset) and 49 during the day (2.5–5.5 h after sunrise). We documented the pregnancy state of these females (Fig. 2A and Suppl. Table 2). The percentage of pregnant females active during either daytime or nighttime did not significantly differ. 63.1 % ± 12.1 % of the females active during the day were pregnant versus 57.7 % ± 17.5 % active at night (mean ± SD for three days + night pairs, GLMM with the reproductive state set as the explanatory parameter, diel activity as the fixed effect, and capture date as the random effect; p = 0.43, df = 79, see Methods and Supp. Table 2.).

### 2.2 Blood measurements

#### 2.2.1 Females’ vitamin D and calcium measurements

**Fig. 3:**
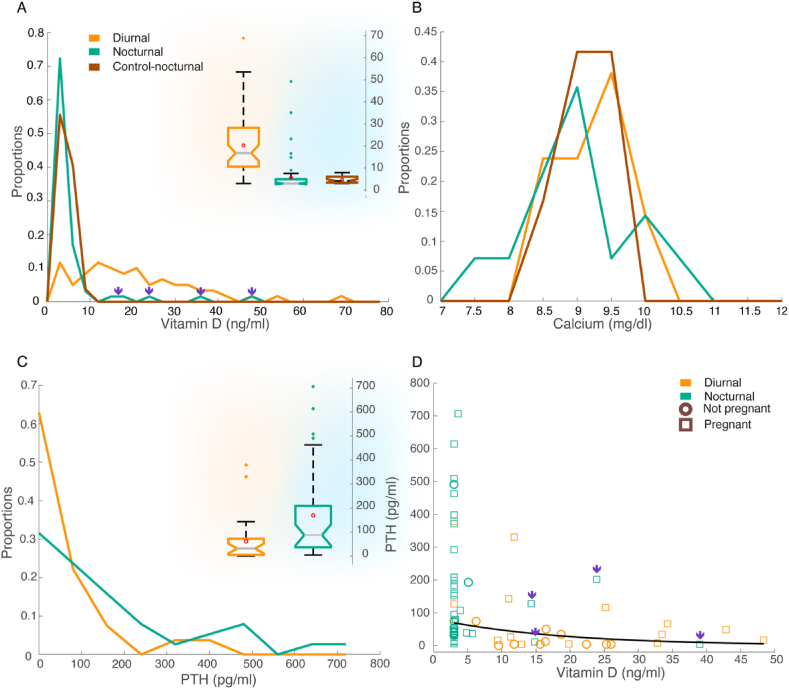
Vitamin D levels are higher in diurnal females while PTH levels are higher in nocturnal females**.** In panels A–C, the proportions on the Y-axis represent the percentage of the total observations that fall within each bin. (A) Vitamin D level distributions in diurnal (orange, n = 61), nocturnal (turquoise, n = 65), and nocturnal control bats (red, n = 27). Insert presents a boxplot of the same measurements: orange shading depicts diurnal activity, and turquoise shading depicts nocturnal activity. Red circles depict the mean values. Purple arrows depict nocturnal bats with high vitamin D levels, probably bats that are active during both daytime and nighttime. **(B)** Calcium level distributions in diurnal (orange, n = 21), nocturnal (turquoise, n = 14), and nocturnal control bats (red, n = 12). (**C)** PTH level distributions in nocturnal (turquoise, n = 38) and diurnal bats (orange, 27). Insert depicts a boxplot of the same measurements, orange shading depicts diurnal activity, and turquoise shading depicts nocturnal activity. Red circles depict the mean values. (**D)** PTH levels decrease when vitamin D levels increase. Each symbol represents one bat and is color-coded according to its activity time: diurnal in orange and nocturnal in turquoise. Circle symbols depict non-pregnant females and rectangles depict pregnant females. Purple arrows depict nocturnal bats with high vitamin D levels, probably bats that are active during both daytime and nighttime. (For interpretation of the references to color in this figure legend, the reader is referred to the Web version of this article.)

Next, we examined the vitamin D levels in diurnal and nocturnal active bats. A total of 251 vitamin D serum blood tests were conducted on 233 individual bats (females and males), from both the DC colony and from a control colony where diurnal activity has never been reported. We excluded those bats that had been caught (more than once) during both daytime and nighttime (see Methods and Suppl. Table 3). We first compared diurnal and nocturnal females. Vitamin D levels were significantly higher in diurnal bats (Fig. 3A, 20.25 ± 13.63 ng/ml, n = 61 diurnal bats, vs. 5.6 ± 7.52 ng/ml n = 65 in nocturnal bats; p = 1.6196 × 10⁻⁸, df = 119; GLMM with vitamin D set as the explanatory parameter, diel activity as the fixed effect, and the lab test day as a random effect). The control group displayed an average vitamin D level of 4.62 ± 1.51 ng/ml (n = 27) which did not significantly differ from the nocturnal DC bats (p = 0.49, df = 90; GLMM with vitamin D set as the explanatory parameter, the colony as a fixed effect and the lab test day as a random effect).

Given vitamin D's role in regulating blood calcium levels, we also measured serum calcium levels in the same three groups (nocturnal, diurnal, and control). Serum calcium levels did not differ significantly between diurnal and nocturnal females and were not correlated with vitamin D levels (Fig. 3B, 9.18 ± 0.57 mg/dl n = 21, vs. 9.03 ± 0.76 mg/dl, n = 14; p = 0.77 for diel activity and, p = 0.19 for the correlation with vitamin D, df = 33; GLMM with calcium levels set as the explanatory parameter, diel activity and vitamin D as fixed effects, and the lab test day as a random effect).

The control group displayed an average calcium level of 9.1 ± 0.32 mg/dl (n = 12), which did not significantly differ from the nocturnal DC bats (p = 0.94, df = 24; GLMM with calcium as the explanatory parameter, colony as the fixed effect, and the lab test day as a random effect).

#### 2.2.2 Males’ vitamin D and calcium measurements

The males exhibited the same vitamin D and calcium patterns as the females. All analyses and statistics used for the males were similarly used for the females (see above and Methods). Diurnal males' vitamin D levels were significantly higher than nocturnal males’ levels (Supp. Fig. S1A, 25.98 ± 21.1 ng/ml n = 23 vs 8.48 ± 14.59 ng/ml, n = 44; p = 0.03. df = 65) The nocturnal control colony displayed an average vitamin D level of 3.34 ± 0.41 ng/ml (n = 7), which did not significantly differ from the nocturnal DC bats (p = 0.33, df = 49). Serum calcium levels did not differ significantly between diurnal and nocturnal males and were not correlated with vitamin D levels (Supp Fig. S1B, 9.29 ± 0.54 mg/dl n = 8, vs. 9.4 ± 0.75 mg/dl, n = 10; p = 0.55 for diel activity and 0.34 for the correlation with vitamin D, df = 15. We excluded the control group from the analysis as we had only 4 males (Suppl. Table 3).

#### 2.2.3 Females’ PTH measurement

In addition to assessing vitamin D and calcium levels, we also measured PTH levels in bats from both the diurnal and nocturnal groups. PTH levels were significantly higher in nocturnal females (Figs. 3C and 168.97 ± 185.52 pg/ml, n = 38 vs. 61.09 ± 93.75 pg/ml, n = 27; p = 0.002, df = 20; GLMM with PTH set as the explanatory parameter, and diel activity, calcium, and pregnancy set as fixed effects; calcium and pregnancy were non-significant with p-values of 0.24 and 0.62, respectively).

There was a significant negative non-linear correlation between PTH and vitamin D levels (n = 65, p = 0.0002, r^2^ = 0.19 - Pearson's linear correlation following a logarithmic transformation, Fig. 3D).

## 3 Discussion

### 3.1 Urban settings and their impact on diurnal activity

In recent years, the increase in urbanization has become a focus of research attention due to its rapid and significant global impacts on species [[56], [57], [58]]. Egyptian fruit bats have expanded their temporal niche and become active during daytime in Israel, mostly in urban environments (Fig. 1A + B and Suppl. Table 1). It has been previously shown that nocturnal Egyptian fruit bats benefit from urban areas because cities provide a richness and diversity of food trees that are close to the bats’ roosting sites [59,60].

We suggest that urbanization also enables fruit bats to cope with the risks entailed by diurnal activity, including predation and hyperthermia. Studies indicating lower predation risks for birds in urban areas [61] suggest a potential parallel reduction in predation risks for fruit bats in urban environments, explaining the diurnal bat activity that is occasionally observed on islands. Bats that are active during the day also face the risk of hyperthermia due to the rising body core temperature during flight [[62], [63]]. In a lab experiment, it has been shown that there is a 2 °C increase in body core temperature in diurnally-active *Carollia perspicillata* bats in comparison to when they fly during the night [64]. Moreover, it has been shown that the diurnally-active lesser sac-winged bat (*Saccopteryx leptura*) on Gorgona Island avoids flying during daytime under higher light intensities and temperatures, to reduce the risk of hyperthermia [17]. Urban areas provide numerous artificial pools where bats can drink, enabling them to cope with the diurnal heat [62,65]. (A total of 11 pools where bats were observed drinking are documented in Suppl. Fig. S2) [42]. The bats dampen their fur and then lick off the water, in a behavior that aids in both hydration and cooling. Evaporation of the water from their fur further facilitates thermoregulation [63].

In contrast, rural bats must travel greater distances to forage [59], often flying in the open (and not amid buildings). Consequently, they may be more vulnerable to predation and have a higher risk of hyperthermia due to longer commutes, prolonged exposure to the sun, and potentially fewer water sources available. This disparity could account for the lack of reported diurnal activity among Egyptian fruit bats in rural areas.

Urbanization however also has negative effects on bats, one of which is anthropogenic noise [65,66]. The DC colony is situated in a shopping center suppliers' parking lot, where the noise from trucks and cars could potentially disturb the bats and push them into foraging during the day. We therefore examined whether the diurnal activity of bats on weekdays differs from their diurnal activity on weekends, when traffic is minimal to none, but did not find any significant difference between them, leading us to believe that the reason for the bats’ diurnal activity is unrelated to noise (see Results and Methods). Furthermore, if noise was the primary factor, we would expect the diurnal activity to be equally spread across both sexes and not predominantly observed in females (see Fig. 2A and C).

### 3.2 Sex-dependent temporal niche shift

Nevertheless, urbanization alone probably cannot account for the diurnal activity of fruit bats in Israel, as our findings indicate that most of the bats' diurnal activity in Tel Aviv is exhibited by female bats, accounting for more than 81 % of the adults’ diurnal activity (Fig. 2A and C).

Mammalian niche shift has primarily been documented at the population level, with both males and females, as well as juveniles, exhibiting temporal niche shifts [5]. Although examples of shifts at the sex level are rare, one such example is that of male *Hipposideros ruber* bats on São Tomé Island, which display diurnal activity [15]. The observed sexual segregation in foraging timing between males, and females could be explained by the predation risk hypothesis, suggesting that despite the greater foraging risk, the new niche is more rewarding (more food abundance) [15,67]. In this case, although by foraging during the day males are at risk of hyperthermia [15,64], it is hypothesized that their diurnal activity is a result of temporal sexual segregation in foraging patterns, with the males exploiting different prey resources to those exploited by the nocturnal females [15]. In the sac-winged bat (*Saccopteryx leptura*) on Gorgona Island, it has been suggested that most of the diurnal activity during the peak season (July) is exhibited by females, particularly those that are pregnant or lactating. Although it has been hypothesized that these females become diurnal to meet their increased energetic demands by foraging on diurnal insects [68], this remains to be quantified [17].

Female Egyptian fruit bats in Israel (and other temperate areas) exhibit two reproduction seasons, giving birth in March and August. The pregnancy lasts four months, followed by a lactation period of about two months [52]. Our research has revealed that it is mostly the female Egyptian fruit bats that engage in daytime foraging, including during pregnancy (November–February and June) and lactation (March-April) [52], emerging even during the hottest hours, exposing themselves to predation risk and the potential threat of hyperthermia (Fig. 1, Fig. 2C). There might be several advantages to such diurnal activity.

### 3.3 Extended foraging periods

Female bats might expand their temporal niche due to the need to forage for extended periods [69,70]. In our study, we recaptured 31 of the 206 RFID-tagged bats (see Methods), with some individuals being recaptured up to five times. Notably, ten of these recaptured bats (32 %) were captured during both day and night across separate events, as also suggested by the high vitamin levels of some of these bats that were captured at night (see purple arrows in Fig. 3A and D, and Suppl. Fig. S1A). While it is unclear whether these bats are active during both day and night on the same day, the fact that bats often left the roost in the middle of the day (Fig. 1B) suggests that they were not simply extending their nocturnal foraging hours.

### 3.4 Diurnal bat diet

Foraging during the day might offer reproductive females access to additional food resources, thus fulfilling their growing energy demands. Egyptian fruit bats’ diet is widely documented in the literature [59,71], and in our study we did not find a discernible difference between the diurnal and nocturnal bats' documented diet (Methods, Suppl. Table 4). However, as we did not quantify the diet of diurnal bats, we cannot exclude the possibility that during pregnancy and lactation diurnal females forage on different fruit trees than males and non-pregnant females. Further research should thus be conducted to address this question.

## 4 Supplying calcium demands during pregnancy

Females undergo a more demanding life cycle, characterized by reproduction and high metabolic demands [37,38], including increased calcium requirements. Calcium is crucial during pregnancy and lactation, as females transfer this essential mineral to their fetuses and newborns, supporting bone development and metabolism [38,39]. In insectivorous bats (*Myotis lucifugus*), lactation-associated bone resorption has been observed [72], and fruit-eating Phyllostomid bats (*Artibeus lituratus*) demonstrated a 22–31 % decrease in bone trabecular area in lactating females [73].

Female bats have evolved various strategies to meet their high energy and calcium demands during pregnancy and lactation. Bats typically have small litter sizes relative to their body mass [33,38]. Insectivorous and frugivorous bats, mostly neotropical but also a few Pteropodidae species [74], have been observed drinking from mineral licks) water sources rich in calcium and other minerals [[74], [75], [76], [77], [78], [79]]). In laboratory experiments, reproductive frugivorous female bats, specifically *Pteropus tonganus*, have been observed licking calcium blocks [80]. Moreover, it has been shown that frugivorous female bats, such as Seba's short-tailed fruit bat (*Carollia perspicillata*), alter their diets during the lactation stage to include fruits that are rich in calcium [81,82]. Additionally, frugivorous bats, including *Pteropus tonganus* and other Pteropodidae species, were observed incorporating calcium-rich leaves into their diets [83]. The diet of Egyptian fruit bats too includes leaves, mostly from *Ficus religiosa*, especially during the winter, which is also a reproduction season [71]. It has been hypothesized that the reason for consuming these leaves is due to the low abundance of fruits during the winter, and the leaves also provide a good nitrogen supplement for these bats [71]. It is currently unknown whether female Egyptian fruit bats consume leaves or to what extent. Egyptian fruit bats' diet also includes pollen [84], and some evidence of consuming insects (*Pachnoda sinuata*) that are more likely a source of protein rather than calcium [85].

It has been shown that Egyptian fruit bat females have been coping with the high energetic demands of reproduction by increasing their food consumption. During gestation, pregnant females’ energy intake was shown to increase by 35 %, while during lactation, the metabolic energy intake was substantially higher, with an 80 % increase compared to non-pregnant females [53]. Despite increasing food consumption it has been shown that Egyptian fruit bat reproductive females do not exhibit a preference for fruits that are particularly high in calcium, e.g. ficus [86]. One possible explanation for this behavior could be that while *Ficus* fruits are indeed rich in calcium, they also contain high levels of oxalate, which might reduce the overall calcium intake from these fruits [76]. Thus, females might acquire calcium in additional ways.

### 4.1 Calcium homeostasis by vitamin D and PTH

Another mechanism for maintaining calcium homeostasis involves the regulation of vitamin D levels [24]. Mammals can obtain vitamin D through their diet or by exposure to sunlight, specifically to UV-B radiation [24]. However, bats' nocturnal activity prevents them from regular exposure to sunlight and UV-B radiation, necessitating alternative means of acquiring vitamin D, such as from their diet, as fish-eating bats do [28]. It has been hypothesized that *Artibeus jamaicensis* bats might obtain vitamin D from the leaves they consume [87], and it was later also suggested that they might obtain it from the insects on which they occasionally forage [28]; however, this remains an open question. As far as we know to date, there is no known dietary source of vitamin D for Egyptian fruit bats [26].

Studies have suggested that bats, which have been nocturnal for millions of years, might have developed passive mechanisms for calcium absorption (vitamin D-independent) similar to the naked mole rat, which utilizes passive intestinal absorption to assimilate calcium [[88], [89], [90], [91]]. However, the evidence indicates that Egyptian fruit bats possess a vitamin D binding protein in their kidneys [92] and it has been shown in a captive experiment that they can synthesize active vitamin D from UV-B radiation after being exposed to the sun for a prolonged period [18,26].

Our current findings match the vitamin D levels reported from previous captive experiments on Egyptian fruit bats [26]. The average vitamin D levels observed in the diurnally-active female bats were 3.5-fold higher than those in the nocturnally-active female bats, with 20.2 ng/ml vs 5.6 ng/ml respectively (Fig. 3A). In males, vitamin D levels were 4.5-fold higher (Supp. Fig. S1A). Because some bats appear to be active by both day and night, the average differences could potentially be even greater.

The parathyroid hormone's (PTH) primary function is to maintain calcium homeostasis in the body [24,31]. When PTH levels increase, the number of mature osteoclasts also increases, leading to bone resorption [24,29], which can result in bone loss. Our results show a negative correlation between vitamin D and PTH levels: thus, when vitamin D levels increase the PTH level decreases and vice versa, as also well described in other mammals [32,93]. It has been previously shown that in the little brown bat (*Myotis lucifugu*) the parathyroid glands are hyperactive during pregnancy and lactation, which could be partially correlated with bone remodeling [35]. PTH and vitamin D levels were also measured in captivity in the straw-colored fruit bat (*Eidolon helvum*)*,* but no correlation was found between them [50].

Our current findings reveal that the mean PTH levels in nocturnal female bats are significantly higher than in diurnal females, with levels more than 2.5-fold higher, approximately 169 vs. 61 pg/ml, respectively (Fig. 3C). We also observed high PTH levels in the nocturnal males (Suppl. Fig. S1C). Our calcium findings are consistent with previous reports for this species [94,95]. It's important to note that there is currently no established PTH serum reference measurements for bats, including *Rousettus*.

### 4.2 Other vitamin D roles

The benefits of vitamin D extend beyond calcium regulation, as studies have shown that vitamin D levels also play a role in immune functioning [[22], [23], [24], [25]] and brain development of rats and mice juveniles [20,21]. The impact of elevated vitamin D levels derived from foraging during the daytime on the bats' immune system or brain development, remains unknown. Further studies should address these questions.

### 4.3 Diurnal activity in juvenile bats

In our study, approximately 24 % of the diurnal activity was performed by juvenile bats. Although we did not find a significant difference from the nocturnal activity of juvenile bats, this percentage is relatively high. The reason why juvenile bats forage during the daytime remains an open question. However, we hypothesize that these young bats learn this behavior from their mothers. It has been shown that nocturnal Egyptian fruit bat pups learn to navigate from their mothers while being carried; and that the females leave their pups on trees while foraging and then return to lactate them (known as "drop-off" behavior, see Ref. [96]). During our study we observed such a drop-off during the day, when at least two females left their pups on a tree in a public garden in Tel Aviv. It has also been shown that during their first independent navigations, nocturnal pups return to the drop-off trees where their mothers had previously left them and even follow the same navigation paths [96]. We hypothesize that diurnal juveniles might also learn the temporal activity patterns from their mothers (in addition to navigation paths), which could lead them to forage during the daytime.

## 5 Future research on calcium homeostasis in bats

Our study revealed a natural elevation of vitamin D levels due to exposure to UV-B radiation in wild diurnally-active bats. This increase in vitamin D levels is negatively correlated with PTH levels; and, to our knowledge, this is the first time this correlation has been directly measured and demonstrated in bats, albeit well-known in other mammals. Despite being nocturnal for millions of years, with little to no exposure to sunlight and, in some species, without a known dietary source of vitamin D, bats have evolved to function well and, with vitamin D levels that would be considered deficient in humans, without affecting their calcium homeostasis [18]. Most studies have focused on vitamin D and calcium levels, rather than PTH [18,87,90]. We suggest that to further our understanding of calcium homeostasis in bats, future research should also focus on PTH.

## 6 Summary

In conclusion, our study has revealed that the temporal niche shift in Egyptian fruit bats is predominantly carried out by females in various stages of reproduction, and by juveniles, in urban settings. Considering the extended reproduction demands on female Egyptian fruit bats, which include four months of pregnancy and 80 days of lactation, and for some females this occurs twice a year, we suggest that Egyptian fruit bat females might derive physiologically beneficial effects from shifting their activity to the daytime, despite the increased risks involved. Although we cannot exclude the possibility that lactating and pregnant females forage during the daytime to meet their energetic demands (such as needing more food or different fruit types), we hypothesize that vitamin D may also play a role in this temporal shift. This could be important not only for calcium homeostasis but also for other benefits of vitamin D, such as immune functioning [[22], [23], [24], [25]] and brain development of the offspring and young bats [20,21]. Further research is needed to fully understand the mechanisms and benefits that elevated vitamin D levels have on these bats.

## 7 Methods

### 7.1 Permits

All experiments were conducted in accordance with the guidelines of the TAU IACUC and approved under permit number TAU-LS-IL-2201-114-2. Bat capture was also approved by the Israel National Park Authority under permit numbers: 2021/42859, 2022/43131.

A. Blood measurements

### 7.2 Animals

Between November 2020 and April 2023, we captured a total of 238 Egyptian fruit bats (*Rousettus aegyptiacus*) from two wild colonies, for the extraction of blood samples (vitamin D, calcium, and PTH). See Suppl. Table 3 for a summary of all the measurements taken from all bats.

### 7.3 Test group capture

We captured 204 male and female bats from a colony located in Tel-Aviv's underground shopping center (the 'Dizengoff Center'). This bat colony is known for its diurnal activity [42]. We captured bats using mist nets and hand nets in the evening (after sunset) and late morning (between 9:00 and 12:00, 2.5–5.5 h after sunrise), sampling both nocturnal and diurnal bats. In the evenings we captured emerging bats, which we defined as nocturnal, while in the mornings we captured both emerging and returning bats, which we defined as diurnal. It has been shown that Egyptian fruit bats in South Africa typically return to their colony approximately 80 min before sunrise [86] and in Israel this takes place during the last hour of the night [53,97]. Thus, by starting to capture bats 2.5 h after sunrise we ensured that no nocturnal individuals were captured.

### 7.4 Nocturnal activity

To validate the typical return of Egyptian fruit bats to their colony, we analyzed the return events of all bats (a total of 2894 events) from December 2022 to May 2023, at our wild bat colony based at Tel Aviv University [98] (Suppl. Fig. 3). The colony entrance is equipped with 24/7 motion-detection cameras (GeoVision Inc. and Imagingsource Inc.), which allows us to precisely monitor the times of each bat's entrance and exit. All entrance events are manually scrutinized. We analyzed all entrances from midnight to the afternoon (17:00) of each day.

### 7.5 Control colony capture

We also captured 34 male and female bats, using hand nets, from a colony located in a rural area in central Israel (‘Tinshemet’ cave), where no diurnal activity has been reported.

### 7.6 Housing

All bats were brought to the lab for blood extraction and were housed in a 4 x 4 × 3.6 m colony under a day/night light cycle, controlled temperature of 26 °C, and provided with fresh fruit *ad libitum* daily. They were marked with an RFID chip (Virbac) and released back into their colonies between 1 and 4 days post capture, except for the control colony bats, which were brought to the lab and released back into the colony immediately after blood extraction.

### 7.7 Blood collection

Blood was collected on the first morning post capture. We collected blood from a total of 238 bats from both colonies (204 from the test group and 34 from the control, Suppl. Table 3). The bats were hand-restrained, without anesthesia. Approximately 1 ml of blood was obtained via venipuncture using micro container separation gel tubes (BD SST™ Serum Tube with Separating Gel, NJ, USA) from the antebrachial vein in the wing. The blood samples were centrifuged at 10k rpm for 3 min to separate the packed cells from the sera. The sera were then collected and sent for analysis (vitamin D and calcium). A portion of it was kept at −20 °C for further analysis (PTH). All individuals were provided with mango juice immediately after handling.

### 7.8 Vitamin D and calcium levels

Lab analyses for vitamin D and calcium were carried out by a veterinary laboratory (American Medical Laboratories (AML), Herzliya, Israel).

### 7.9 Vitamin D

Individual serum was analyzed for Vitamin D (25-OH) level using the Elecsys Vitamin D total III assay (Roche Diagnostics, GmbH, Mannheim, Germany) as described in Ref. [99]. This assay is based on the competition principle. It employs a vitamin D binding protein labeled with a ruthenium complex as a capture protein to bind 25-hydroxyvitamin D3 and 25-hydroxyvitamin D2. Cross-reactivity to 24,25-dihydroxyvitamin D is blocked by a specific monoclonal antibody.

The assay is based on three different incubation phases. The reaction mixture is then aspirated into the measuring cell and magnetically captured onto the surface of an electrode. Application of a voltage to the electrode induces chemiluminescent emission, which is measured by a photomultiplier and later calibrated via a calibration curve to determine the results.

A total of 251 sera were analyzed for vitamin D (25-OH) level. From some of the bats (n = 14) we collected blood twice post recapture. For these bats, we present their average results (a total of seven blood samples). Bats that were (re)captured both during daytime and nighttime (n = 4 bats, a total of nine blood samples) were excluded from the comparison.

Recent years have seen an ongoing debate regarding the correct assessment of vitamin D levels. Some argue that measuring total vitamin D, as done here, is sufficient, while others suggest that a more accurate assessment involves measuring free 25(OH)D [93,100,101]. However, we also measured parathyroid hormone (PTH), which is negatively correlated with 25(OH)D. Studies to date have not shown a significant difference between the correlation of PTH with total 25(OH)D versus free 25(OH)D [93]. It thus appears that although there might be no advantage to measuring free 25(OH)D over a total 25(OH)D in bats, it would be interesting to conduct further measurements [100].

### 7.10 Calcium

We also measured the calcium levels for 69 bats for which we had sufficient serum. Individual serum was analyzed using CA2 calcium Gen 2 (Roche Diagnostics, GmbH, Mannheim, Germany, as an *in-vitro* test, for the quantitative determination of calcium in human serum plasma and in urine. In this test, the calcium ions in the sera react with 5-nitro-5′-methyl-BAPTA (NM-BAPTA) under alkaline conditions to form a complex. This complex reacts in the second step with EDTA. The change in absorbance is directly proportional to the calcium concentration and is measured photometrically to calculate the analyte concentration of each sample. For more details regarding this assay see Ref. [102].

### 7.11 PTH levels

The bone metabolism multiplex assay MILLIPLEX® Human Bone Magnetic Bead Panel (HBNMAG-51K) from Merck KGaA, Darmstadt, Germany, was used to assess PTH (parathyroid hormone) levels in serum samples from both nocturnally-active bats and diurnally-active bats in the test group. The assay was performed according to the manufacturer's protocol, using two 96-well plates that contained a lyophilized standard cocktail and two quality controls, allowing for duplicate measurements of up to 74 serum samples (42 nocturnally-active bats and 32 diurnally-active bats; one of the bats was tested twice, providing its mean result). A 1:2 dilution of 25 μg of serum was incubated with antibody-conjugated beads overnight at 4 °C. A biotinylated detection antibody was added to the bead complexes and kept on a plate shaker for 30 min, followed by incubation with streptavidin-phycoerythrin for an additional 30 min. After washing the wells three times, 100 μL of Sheath Fluid was added to all wells, and the plate was read on a Luminex® 200TM run plate and analyzed using MAGPIX® with xPONENT® software.

B. Population assessment

### 7.12 Sex and age ratio activity evaluation

In addition to the above-noted captures for the blood samples, to determine the sex and age ratio of diurnally-active Egyptian fruit bats in the DC colony, we conducted ten sequential days and nights of bat captures using mist nets (same method as above, Suppl. Table 2). The captured bats’ sex and age (adult/juvenile/independent pup) were noted. In our analysis, we differentiated between adult bats, categorizing them by sex, and juvenile bats, which were combined into a single group irrespective of sex. We also tagged bats from which we had extracted blood, using RFID tags. We used an RFID scanner to scan all bats for recapture identification.

## 8 Reproduction state assessment

In addition, to determine ratio of pregnant females of diurnally-active Egyptian fruit bats in the DC colony, we conducted six sequential days and nights of bat captures using mist nets (same method as above, Suppl. Table 2). Pregnancy assessment was carried out by gently pressing and gauging the size of the uterus. To ensure consistency, the pregnancy assessment was always performed by the same experimenter – a highly experienced veterinarian specializing in bats.

C. Diurnal activity assessment

### 8.1 Diurnal bats' emergence time from the colony

During the summer of 2020 we conducted observational studies (related to another study [42]) on the emergence patterns of the DC bat colony. Observations were made over a total of 21 days, from 10:00 to 15:00 (sunset was at ∼19:00). A total of 604 bat emergences were documented. In instances of incomplete hourly observations, the data were adjusted to reflect the mean emergence rate per hour (with the exception of observations of 20 min or less, which were excluded entirely). All data were systematically logged into the Epicollect5 application [103].

### 8.2 Surveying diurnal bat distribution in Israel

In recent years, there has been a growing use of citizen-science by researchers to assess animal activity and distribution [[104], [105], [106], [107], [108]]. Since the autumn of 2020 we have conducted a citizen-science survey to track the diurnal activity of fruit bats across the country, using a method also used in Speakman [[104], [105], [106]]. The ongoing survey is operated by the citizen-science department of the Society for the Protection of Nature in Israel, using the ArcGIS website platform (https://survey123.arcgis.com/share/fce89cc471b14a7486626b2574fd4b73). We promoted the survey through various news outlets, including television and major news websites, as well as on social media platforms, periodically throughout the year. We encouraged citizens to engage by filling out an online survey form in which they could submit detailed observations of diurnal bats. The information requested included the location, date, time, and number of bats sighted, the type of tree observed, and any accompanying photographs of the bats. We received more than 470 reports. We selected only the validated observations (a total of 322 observations of 432 bats). Our validation criteria comprised: 1. reports with accompanying photos (a total of 91 observations). or 2. reports from experienced bat researchers and skilled birders capable of identifying flying fruit bats (a total of 159 observations); and 3. observations made in proximity to known bat daytime activity areas (a total of 72 observations). We define proximity as a maximum of 500 m from a confirmed observation (photo/zoologist/birder, or proximity to a known bat colony (e.g. DC)). The mean proximity was 101 ± 167.45 m (mean ± SD). We defined daytime activity as occurring between 1 h after sunrise and 1 h before sunset.

### 8.3 Diurnal bats’ diet

To investigate the foraging behavior of diurnal bats, we conducted extensive monitoring in Tel Aviv, from the spring of 2019 to the spring of 2023, resulting in over 10,000 photographs documenting the diurnal bats’ foraging habits. These photographs were taken by a wildlife photographer across various locations in Tel Aviv. We supplemented this dataset with images obtained from observations of diurnal bats throughout Israel, sourced from the citizen-science survey, with a total of 65 different observers. From analysis of the photos, we were able to identify the specific fruit trees on which the bats were foraging of (see Suppl. Table 4)

D. Statistical analysis

All the analyses were performed using Matlab 2024 (Mathwork).

To determine the emergence hour and day type (weekend or weekday), we used a mixed-effect generalized linear model (GLMM) with the number of observations as the explanatory parameter, the hour of activity and day type as fixed effects, and the date as a random effect.

To determine the sex dependency of diel activity, we used a mixed-effect generalized linear model (GLMM) with a logit link function, with sex as the explanatory parameter, activity time (day/night) as a fixed effect, and the capture date as a random effect.

To determine the age dependency of diel activity, we used a mixed-effect generalized linear model (GLMM) with a logit link function, with age as the explanatory parameter, diel activity as a fixed effect, and the capture date as a random effect.

To determine the reproductive state dependency of diel activity we used a mixed-effect generalized linear model (GLMM) with a logit link function, with the reproductive state set as the explanatory parameter, and diel activity as the fixed effect.

To determine the relation between vitamin D level and diel activity we used a mixed-effect generalized linear model (GLMM) with vitamin D as the explanatory parameter, diel activity as a fixed effect, and the lab test day as a random effect to account for potential batch effects.

To determine the difference between the DC colony and the control colony in vitamin D levels we used a mixed-effect generalized linear model (GLMM), with vitamin D as the explanatory parameter, colony as a fixed effect, and the lab test day as a random effect to account for potential batch effects.

To determine the relation between calcium level with diel activity and vitamin D levels, we used a mixed-effect generalized linear model (GLMM), with calcium as the explanatory parameter, and diel activity and vitamin D levels as fixed effects, and the lab test day as a random effect to account for potential batch effects.

To determine the difference between the DC colony and the control colony in calcium levels we used a mixed-effect generalized linear model (GLMM), with calcium as the explanatory parameter, colony as a fixed effect, and the lab test day as a random effect to account for potential batch effects.

To examine the correlation between PTH and daily activity in bats, we took into consideration the pregnancy state of the bats (pregnant or not pregnant) and assessed its potential impact on the outcomes. We ran a generalized linear model (GLMM), with PTH as the explanatory parameter, and diel activity, calcium, and pregnancy as fixed effects. We included pregnancy in this model because PTH is known to be affected by it in other mammals [109].

To determine the correlation between PTH and vitamin D[110], we converted the PTH and vitamin D levels to logarithmic scales and ran a Pearson's linear correlation test.

## Data availability

All data and materials are available from https://data.mendeley.com/datasets/7bw67mv9jb/1.

## Funding

The first author (OE) was partially funded by Dr. Alexander Lester and Eva Lester fellowships.

## CRediT authorship contribution statement

**Ofri Eitan:** Writing – review & editing, Writing – original draft, Visualization, Validation, Software, Project administration, Methodology, Investigation, Formal analysis, Data curation, Conceptualization. **Maya Weinberg:** Writing – review & editing, Writing – original draft, Validation, Methodology, Investigation, Data curation, Conceptualization. **Nirit Lavie Alon:** Writing – review & editing, Resources. **Sahar Hiram-Bab:** Writing – review & editing, Investigation, Data curation. **Yuval Barkai:** Writing – review & editing, Resources, Data curation. **Reut Assa:** Writing – review & editing, Formal analysis, Data curation. **Adi Rachum:** Resources, Data curation. **Omer Yinon:** Writing – review & editing, Conceptualization. **Yossi Yovel:** Writing – review & editing, Writing – original draft, Validation, Supervision, Resources, Project administration, Methodology, Investigation, Conceptualization.

## Declaration of competing interest

The authors declare that they have no known competing financial interests or personal relationships that could have appeared to influence the work reported in this paper.
