# Supplementary material for: Elevated vitamin D levels in diurnally-active female fruit bats
Source: Heliyon. 2024 Oct 4;10(20):e38973. doi: 10.1016/j.heliyon.2024.e38973 (PMC11497383; doi:10.1016/j.heliyon.2024.e38973)
Supplement: Multimedia component 1 [file mmc1.docx]

**Elevated vitamin D levels in diurnally-active female fruit bats**

## Supplementary

| **District** | **City** | **Observations** |
| --- | --- | --- |
| Tel Aviv | Tel Aviv | 261 |
| Tel Aviv | Giv'atayim | 9 |
| Tel Aviv | Herzliya | 1 |
| Tel Aviv | Holon | 2 |
| Tel Aviv | Ramat Gan | 21 |
| Southern | Ashdod | 9 |
| Jerusalem | Jerusalem | 1 |
| Haifa | Haifa | 1 |
| Central | Modi’in | 2 |
| Central | Netanya | 1 |
| Central | Petah Tikva | 8 |
| Central | Yavne | 2 |
| Central | Kfar Sava | 1 |
| Central | Lod | 1 |
| Central | Kfar Shmuel | 1 |
| Central | Ness Ziona | 1 |

Table S1: Diurnal activity distribution. Observations made through citizen-science have revealed that the diurnal activity of Egyptian fruit bats is not limited to Tel Aviv but extends to other urban environments throughout Israel.

| **Date** | **Sex/Age** | **Activity** | | **Female reproduction state** | | | |
| --- | --- | --- | --- | --- | --- | --- | --- |
|  |  | **Diurnal** | **Nocturnal** | **Diurnal** | | **Nocturnal** | |
|  |  |  |  | **Pregnant** | **Not**  **pregnant** | **Pregnant** | **Not**  **pregnant** |
| **18-19.11.22** | Adult females | 17 | 9 | 9 | 8 | 7 | 2 |
| **19/25.2.22** | Adult females | 15 | 12 | 9 | 6 | 6 | 6 |
| **27-28.1.23** | Adult males | 4 | 42 | - | - | - | - |
|  | Adult females | 35 | 13 | - | - | - | - |
|  | Juveniles | 0 | 8 | - | - | - | - |
| **10-11.2.23** | Adult males | 3 | 34 | - | - | - | - |
|  | Adult females | 17 | 11 | 13 | 4 | 5 | 6 |
|  | Juveniles | 14 | 6 | - | - | - | - |
| **21-22.4.23** | Adult males | 4 | 8 | - | - | - | - |
|  | Adult females | 24 | 20 | - | - | - | - |
|  | Juveniles | 6 | 1 | - | - | - | - |
| **19-20.5.23** | Adult males | 5 | 16 | - | - | - | - |
|  | Adult females | 11 | 57 | - | - | - | - |
|  | Juveniles | 4 | 12 | - | - | - | - |
| **23-24.6.23** | Adult males | 4 | 14 | - | - | - | - |
|  | Adult females | 13 | 24 | - | - | - | - |
|  | Juveniles | 12 | 9 | - | - | - | - |

Table S2 –Bats' sex and age, and female reproduction state. A summary of the age and sex of the captured bats, alongside assessments of the females’ reproductive state, taken during seven consecutive days and nights, Diurnal activities are highlighted in orange, and nocturnal activities in turquoise. Note that not every parameter was assessed for each separate 24-hour period.

| **Diel Activity** | **Colony** | **Vitamin D** | | **Calcium** | | **PTH** | |
| --- | --- | --- | --- | --- | --- | --- | --- |
|  |  | **Males** | **Females** | **Males** | **Females** | **Males** | **Females** |
| Diurnal | Dizengoff Center (DC) | 23 | 61 | 8 | 21 | 5 | 27 |
| Nocturnal | Dizengoff Center (DC) | 44 | 65 | 10 | 24 | 4 | 38 |
| Nocturnal - Control | Tinshemet | 7 | 27 | 4 | 12 | -- | -- |

Table S3 – Total counts for each measured parameter for all bats combined**.** A summary of the measurements taken from all bats from the DC colony and the nocturnal control colony (“Tinshemet”).

| **Trees Species** |
| --- |
| *Bombax ceiba* |
| *Callistemon phoeniceus* |
| *Celtis australis* |
| *Ceratonia siliqua* |
| *Eriobotrya japonica* |
| *Erythrina corallodendron* |
| *Ficus* |
| *Ficus carica* |
| *Ficus lyrata* |
| *Ficus microcarpa* |
| *Ficus religiosa* |
| *Ficus rubiginosa* |
| *Ficus sycomorus* |
| *Melia azedarach* |
| *Morus alba* |
| *Phoenix dactylifera* |
| *Washingtonia filifera* |

Table S4 – Fruit-tree species comprising the diurnal bats’ diet. A summary of all fruit-tree species observed to be consumed by diurnal bats while foraging. The observed trees do not differ from the tree species previously reported for nocturnal bats^58,70^.


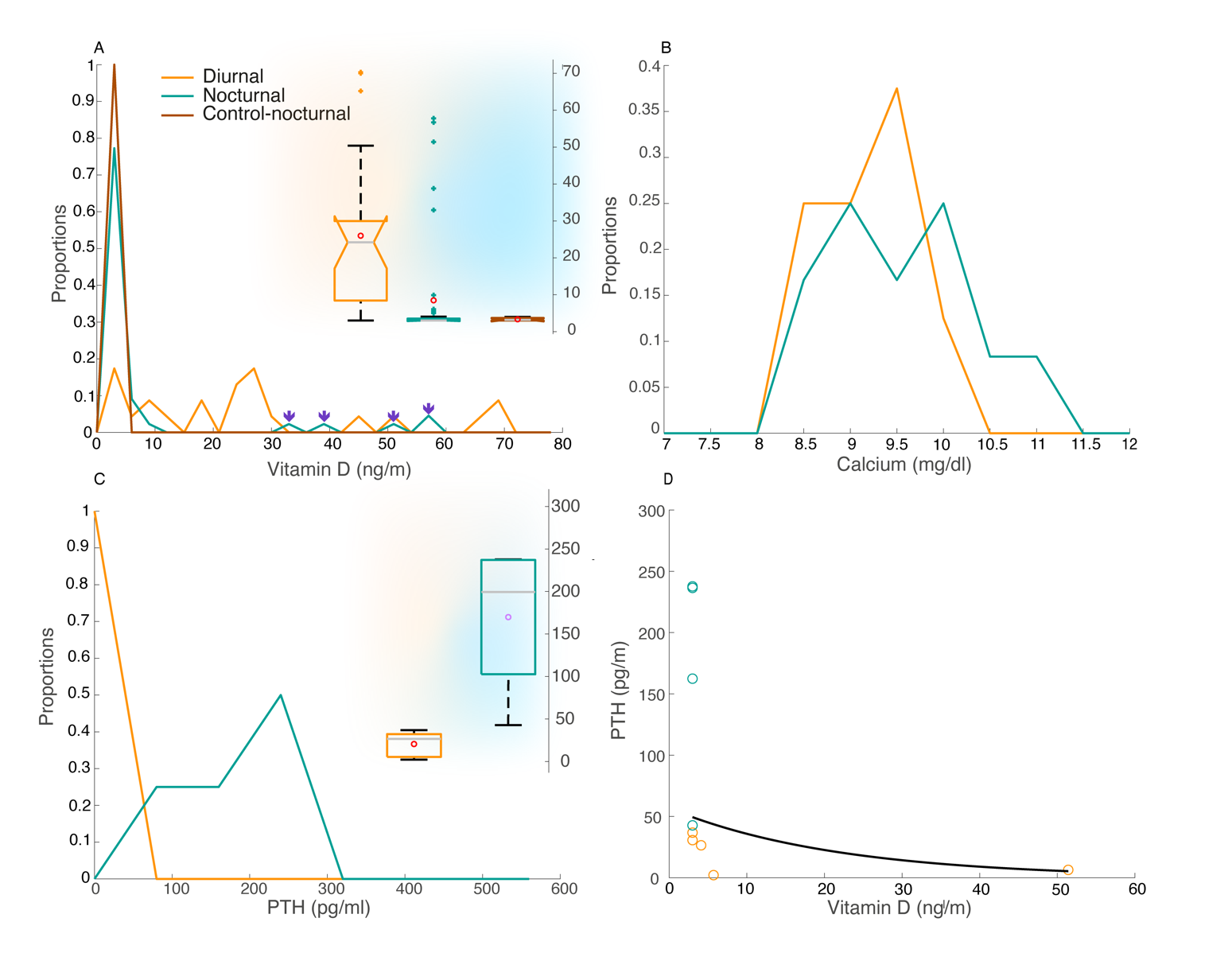


Figure S1- Male bats presented the same patterns as females. In panels A-C, the proportions on the Y-axis represent the percentage of the total observations that fall within each bin. (A) Vitamin D levels distribution of diurnal (orange, n = 23), nocturnal (turquoise, n = 44), and nocturnal control bats (red, n = 7). Insert depicts a boxplot of the measurements, and red circles depict the mean values. Purple arrows depict nocturnal bats with high vitamin D levels, probably those that are active both during daytime and nighttime. Orange shading depicts diurnal activity, and turquoise shading depicts nocturnal activity (B) Calcium level distribution in diurnal (orange, n = 8), and nocturnal (turquoise, n = 10). (C) PTH level distribution in nocturnal (orange, n = 4) and diurnal bats (turquoise, 5). Insert depicts a boxplot of the measurements, and red circles depict the mean values. Orange shading depicts diurnal activity, and turquoise shading depicts nocturnal activity (D) PTH levels decrease when vitamin D levels increase. Each dot represents one bat and is color-coded according to its activity period.

**
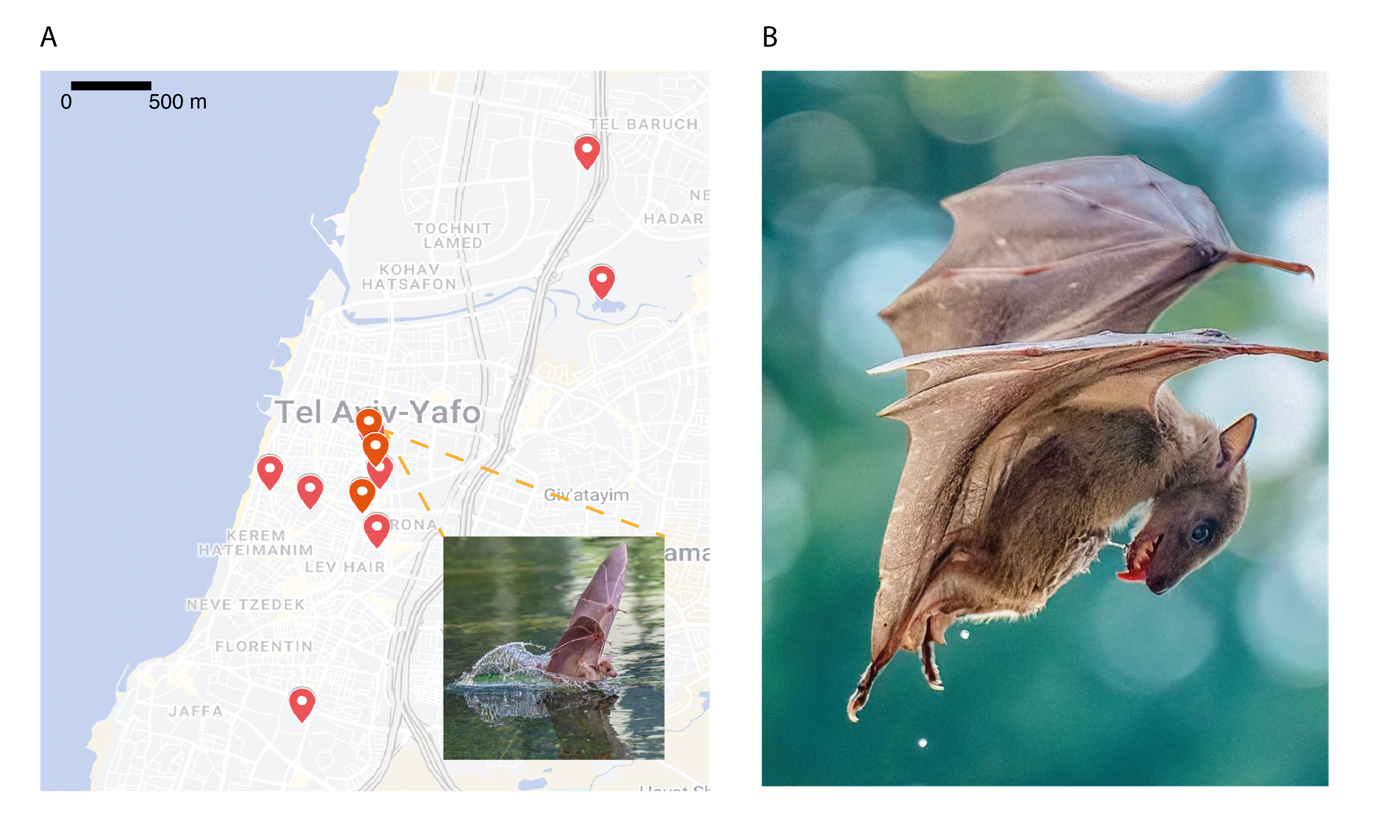
**

Figure S2 – Bats thermoregulate in Tel Aviv by soaking their fur and drinking in artificial pools (A**)**. Locations of artificial pools in Tel Aviv where bats were observed drinking. (B). The bats lick their fur after soaking it in the water.


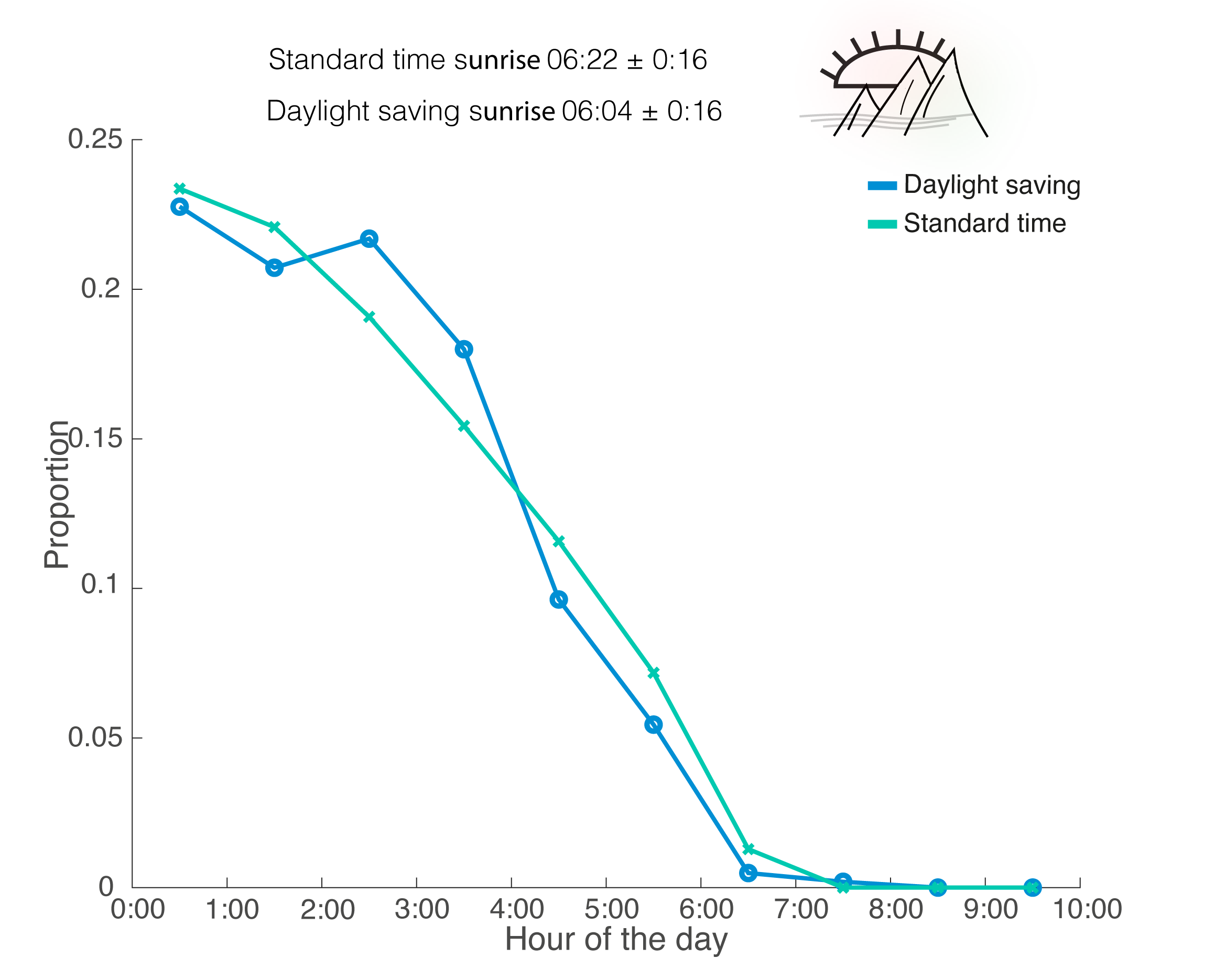


Figure S3 – Bats return to the colony by sunrise. The typical foraging behavior of Egyptian fruit bats involves returning from foraging by sunrise. The blue line represents daylight-saving time, and the turquoise line represents standard time (non-daylight-saving time). Proportions on the Y-axis represent the percentage of the total observations that fall within each bin. The sunrise and sunset times are presented as the mean ± SD for the study period.
